# Reliability and validity of the English, Chinese, Korean, Indonesian, and Vietnamese versions of the public health research foundation stress checklist short form

**DOI:** 10.1186/s13030-023-00271-8

**Published:** 2023-04-06

**Authors:** Yoko HAYASHI, Yoshie IMAZU, Sixin DENG, Masato MURAKAMI

**Affiliations:** 1 Institute of Stress Science, Public Health Research Foundation, 1-1-7 Nishi-waseda, Shinjuku-ku, Tokyo, 169-0051 Japan; 2 Graduate School of Letters, Arts, and Sciences, Waseda University, 1-24-1 Toyama, Shinjuku-ku, Tokyo, 162-8644 Japan; 3 School of Psychology and Medical Management, International University of Health and Welfare, 4-1-26 Akasaka, Minato-ku, Tokyo, 107-8402 Japan; 4 Department of Psychosomatic Internal Medicine, Sanno Hospital, 8-10-16 Akasaka, Minato-ku, Tokyo, 107-0052 Japan

**Keywords:** Stress checklist, Public health research foundation stress checklist short form, Multilingual scale, Reliability, Validity

## Abstract

**Background:**

Foreign nationals residing in Japan account for approximately 2% of the total population (i.e., approximately 2.6 million people). Of these, 12% are not proficient in speaking Japanese and 25% experience difficulty reading Japanese. Therefore, a simple, convenient, and accurate scale in the native language of foreign nationals is required to support their mental health. In this study, the Public Health Research Foundation Stress Checklist Short Form (PHRF-SCL (SF)) was translated into five languages and the reliability and validity of the translations were confirmed.

**Methods:**

The five translated versions of the PHRF-SCL (SF) have been reverse-translated into the original language, Japanese. The creator confirmed that there were no inconsistencies between the Japanese and reverse-translated versions. A total of 777 adults aged 18–64 years participated in the study. They were asked to complete the native language versions of the PHRF-SCL (SF) and Depression Anxiety Stress Scale 21 (DASS 21) online.

**Results:**

An exploratory factor analysis yielded the same four-factor structure as the original. Internal consistency was confirmed by the alpha coefficients of the subscales. Participants were classified into two groups on the basis of the severity classification obtained from each subscale of the DASS 21. Scores of PHRF-SCL (SF) are significantly higher in groups classified as symptomatic by DASS 21, thereby confirming construct validity. Concomitant validity was confirmed based on correlations with the DASS 21.

**Conclusions:**

English, Chinese, Korean, Indonesian, and Vietnamese versions of the PHRF-SCL(SF) have been prepared. Although these versions are subject to further statistical analysis, the results were sufficiently substantiated for practical use. This scale is expected to contribute to the promotion of mental health services for people from these countries.

## Background

Globally, the number of international migrants has been increasing in recent years, which makes it important to insure that mental health measures are available to all residents. According to the Comprehensive Mental Health Action Plan 2013–2030 of the World Health Organization (WHO), “determinants of mental health and mental disorders include not only individual attributes, such as the ability to manage one’s thoughts, emotions, behaviors, and interactions with others, but also social, cultural, economic, political and environmental factors, such as national policies, social protections, living standards, working conditions, and community social supports.” The WHO further describes its vision of the action plan as being “a world in which mental health is valued, promoted and protected, [and] mental disorders are prevented [1].”

People residing in foreign countries experience numerous stressors, including those related to the economy and the environment. Differences in social customs and culture become another source of stress. In addition to the adoption of measures for eliminating mental health risk factors, systems that provide support similar to, or better than, that provided to regional people are necessary to promote mental health and prevent mental disorders among foreign nationals.

There are approximately 2.6 million foreign nationals residing in Japan, comprising roughly 2% of the total population [2]. According to the FY2020 Basic Survey Report on Foreign Residents published by the Immigration Services Agency, 18.7% of foreign residents responded that they could “only give basic greetings” or “barely speak any Japanese.” Alternatively, in terms of Japanese-language reading ability, 25.1% responded that they “can read some basic Japanese,” “can barely read” Japanese, or “cannot read” Japanese [3]. Such individuals are at risk of losing access to appropriate support owing to their limited Japanese-language ability.

Assessing the stress conditions of individuals enables the self-cognition of mental health disorders at an early stage and the undertaking of appropriate intervention. However, an accurate assessment cannot be expected if the individual being assessed does not completely comprehend the questions. Having the tools to assess the mental health of all residents is essential to realize WHO’s vision of a world where mental health is valued, promoted, and protected. Based on this premise, we translated the Public Health Research Foundation Stress Checklist Short Form (PHRF-SCL (SF)) into multiple languages for international use. The PHRF-SCL (SF) is a stress response scale comprising four subscales (autonomic symptoms, fatigue and physical symptoms, feelings of anxiety and uncertainty, and feelings of depression and inadequacy). The reliability and validity of the scale have been confirmed by studies involving more than 30,000 Japanese individuals aged 18 to 64 years [4, 5]. This scale measures stress reactions multidimensionally and concisely with a small number of questions. It can be used to assess the mental health of healthy individuals as, rather than screening for pathologies, it questions respondents about the psychological and physical stress responses that anyone might experience on a daily basis. It allows for the assessment of stress reactions in subjects who are unaware that they are under highly stressful conditions.

In this study, we created translated versions of the PHRF-SCL (SF) for use as a tool for providing mental health services to foreign residents in Japan and examined the reliability and validity of the translated versions. The scale was translated into English, Chinese, Vietnamese, Indonesian, and Korean. The translated scale is expected to provide people using these languages with a simple and multifaceted assessment of psychosomatic stress responses.

## Methods

### Procedure and participants

Originally created in Japanese, the PHRF-SCL (SF) has been translated into five languages. The English-language version was translated by a Japanese psychologist fluent in English, whereas the Chinese and Korean versions were assigned to psychologists who are native speakers of each language and are fluent in Japanese. The Indonesian and Vietnamese versions were both prepared by native speakers of each language who are also proficient Japanese speakers. The scale was reverse-translated into Japanese by a person other than the original translator who was fluent in Japanese and whose native language was the language in question. The retranslation was used to confirm that the questions used expressions that were common to each language. Based on these reverse-translations, the authors of PHRF-SCL (SF) confirmed that there were no inconsistencies with the content of the original version.

Study participants included 777 native speakers of the relevant languages aged 18–64 (385 male, 389 female, three other; mean age: 37.15 ± 14.21). All surveys were conducted online. The services of Cross Marketing Inc. were used to conduct surveys in countries where each language is an official language. The English-language, Chinese-language, Korean-language, Indonesian-language, and Vietnamese-language surveys were conducted in the US, the People’s Republic of China, the Republic of Korea, the Republic of Indonesia, and the Socialist Republic of Vietnam, respectively. However, 77 international students residing in Japan were also included in the Chinese study. The surveys outside of Japan continued to be conducted for each language until there were roughly similar numbers of people in each age group. The surveys by Cross Marketing Inc. were conducted from November 2021 to February 2022, and the survey targeting international students in Japan was conducted from October 2020 to September 2021.

### Measurements

Survey participants were instructed to indicate their native language, gender (male, female, and other), and age (Table 1).

The Depression Anxiety Stress Scale (DASS), developed by Lovibond and Lovibond [6], comprises 42 items for the simple evaluation of “depressive symptoms,” “anxiety symptoms,” and “tension/stress.” The severity of each subscale can be classified into five levels (normal, mild, moderate, severe, or extremely severe) based on the raw scores in each [6]. The DASS 21 is an abbreviated version of the DASS; it is a four-level scale comprising 21 items. In this study, to examine the validity of the PHRF-SCL (SF), we used the original English-language, Korean, Indonesian, and Vietnamese versions as translations from the DASS developer’s website [7]. For the Chinese version, we used Li et al.’s [8] translated version.

**Table 1:** Number and average age of participants in each language version

	English	Chinese	Korean	Indonesian	Vietnamese
Total	150	177	150	150	150
Male	74	87	74	75	75
Female	75	89	75	75	75
Other	1	1	1	0	0
Age (yr)					
18–19	17	19	13	19	17
20–29	30	79	40	32	34
30–39	25	19	27	30	43
40–49	30	22	27	28	29
50–59	25	20	25	27	17
60–64	23	18	18	14	10
Mean(yr) (SD)	39.79 (15.17)	34.26 (14.00)	38.28 (14.24)	37.99 (14.24)	35.93 (12.82)

### Statistical analysis

An exploratory factor analysis of the 24 PHRF-SCL (SF) items was conducted using the maximum likelihood method and promax rotation, and equality between languages was confirmed with the Levene test. A confirmatory factor analysis was conducted on the obtained factor structure to determine goodness of fit. Cronbach’s alpha coefficient was used to check the internal consistency of the subscales. Participants were classified into two groups based on the severity classification obtained from the DASS 21: the “Normal” group and the “Symptomatic” group (mild, moderate, severe, or extremely severe). A *t*-test was conducted to examine construct validity, with the groups (normal or symptomatic) set as the independent variable and the total raw score in the relevant language version of the PHRF-SCL (SF) considered the dependent variable. To verify the concurrent validity, the correlation coefficient between the subscale and total scores of the PHRF SCL (SF) and DASS 21 was determined. SPSS version 23 and Amos 23.0.0 were used for the analysis.

## Results

The 24 items of PHRF-SC(SF) were examined by a maximum likelihood factor analysis with Promax rotation. The analysis produced a solution of four factors theoretically and based on possible interpretations (Table 2).

**Table 2:** Exploratory Factor Analysis of PHRF-SCL(SF)

PHRF-SCL(SF) Items	Factor loading					
	I	II	III	IV		Communalities
**I. Feelings of Anxiety and Uncertainly alpha = .87**						
Whenever I do any work, I do not feel confident about it	**.882**	− .117	− .089	.067		.619
Whenever I do something, I feel anxious that it might not go well	**.754**	.025	.079	− .077		.604
I am not proactive when it comes to getting things done	**.750**	− .048	.035	− .006		.541
When I have to make decisions, I am lost and cannot make up my mind	**.686**	.104	− .020	− .016		.549
I feel anxious about whether I will be able to handle changes in my environment and do my job well	**.639**	.201	− .047	− .025		.561
I feel overwhelmed by the burden of my work duties	**.343**	.280	.038	.061		.431
**II. Feelings of Depression and Inadequacy alpha = .82**						
Sometimes I find it difficult to be around people who are not like-minded	.019	**.725**	− .098	.038		.482
I wish I had someone who would give my efforts their due credit	− .063	**.683**	.096	− .090		.429
I sometimes find it difficult to trust people	.083	**.587**	.150	.026		.133
I can get angry or irritated at the slightest thing	.144	**.533**	− .022	.046		.439
I can be grumpy at times	.143	**.460**	.125	− .009		.445
I sometimes have no hope for the future	**.330**	**.402**	− .064	.077		.477
**III. Fatigue and Physical Symptoms alpha = .83**						
Sometimes my shoulders get stiff or my neck muscles get tight	− .086	.036	**.802**	− .108		.489
I feel drained and cannot relax properly	− .044	.098	**.654**	.081		.571
Sometimes I get a pain in my back or hips	.019	.006	**.641**	.010		.443
I tend to experience eyestrain	.145	.019	**.397**	.081		.345
I get tired easily when doing some activities	.329	.028	**.394**	− .003		.474
I feel woozy and my head feels heavy	.331	− .082	**.341**	.163		.476
**IV. Autonomic Symptoms alpha = .75**						
I sometimes have a pain in my chest	− .106	.084	− .061	**.765**		.495
I’m worried about my heart palpitations	.154	− .094	− .107	**.638**		.386
I am feeling dizzy at times	− .065	− .009	.179	**.521**		.371
Breathing freely suddenly becomes difficult	.012	.050	.086	**.483**		.349
I don’t feel like eating even my favorite foods	.088	− .006	.155	**.356**		.295
I have trouble falling asleep and find it difficult to have a good night’s sleep	.018	.000	**.441**	.137		.315

The extraction method were maximum likelihood method and promax rotation. Factor loadings above 0.30 are in bold.

The results of the exploratory factor analysis are presented in Table 3. The same four-factor structure as the original was obtained. All items had loadings of 0.3 or higher. Unlike the original, the factor loading for the item “difficulty falling asleep and staying asleep” was lower for “autonomic symptoms (.137)” than for “fatigue physical reactions (.441).” However, it was included in “fatigue and physical reactions” in accordance with the original, resulting in a four-factor, six-item structure. Furthermore, confirmatory factor analysis using the above four-factor structure was performed to examine the goodness of the fit. Equivariance of language groups in each factor was established by the Leven test with a risk ratio of 0.05 or greater. Goodness-of-fit indices were χ^2^ = 668.363, df = 246, *p* < 0.001, GFI = 0.930, AGFI = 0.915, RMSEA = 0.047. The alpha coefficient range (0.75–0.87) indicates sufficient internal consistency.

**Table 3:** Inter-factor Correlation

Factor	I	II	III	IV
I	1	.743	.702	.693
II		1	.719	.611
III			1	.714
IV				1

Participants were divided into two groups, normal and symptomatic, on the basis of their scores by severity classification in each subscale of the DASS 21. A *t*-test was performed using these groups as the independent variable and the total raw score of PHRF-SCL (SF) as the dependent variable (Table 4). The PHRF-SCL (SF) scores were significantly lower in the normal groups than they were in the symptomaticgroups, indicating construct validity.

**Table 4:** Comparison of PHRF-SCL (SF) scores between symptomatic and normal groups classified by DASS 21

DASS 21	Group	n	M	SD		t-value	*p*	
Depression	Symptomatic	89	22.20	(9.57)		9.26	< .001	
	Normal	61	8.77	(7.33)				
Anxiety	Symptomatic	86	22.92	(9.21)		10.61	< .001	
	Normal	64	8.44	(6.80)				
Stress	Symptomatic	62	23.90	(10.27)		8.05	< .001	
	Normal	88	11.69	(8.27)				

Symptomatic: DASS 21 subscale scores classified as “mild”, “moderate”, severe,” or “extremely severe.” Normal: DASS 21 subscale scores classified as “normal.”

We determined the correlation coefficients between the PHRF-SCL (SF) subscales and total scores as well as the DASS 21 subscales and total scores. Table 5 shows the correlational analysis in each language. All language versions revealed significantly positive correlations (*p* < 0.001), indicating sufficient concurrent validity.

**Table 5:** Correlation coefficients between each subscale of PHRF-SCL (SF) and DASS 21

		PHRF-SCL (SF)					DASS 21			
		FAU	FDI	FPS	AS	Total	Depression	Anxiety	Stress	Total
PHRF-SCL (SF)	FAU	1	.714*	.690*	.621*	.886*	.627*	.606*	.669*	.672*
	FDI		1	.677*	.573*	.865*	.600*	.551*	.638*	.633*
	FPS			1	.678*	.883*	.554*	.551*	.608*	.605*
	AS				1	.814*	.592*	.658*	.632*	.663*
	Total					1	.687*	.681*	.737*	.744*
DASS 21	Depression						1	.814*	.848*	.943*
	Anxiety							1	.846*	.936*
	Stress								1	.952*
	Total									1

FAU: Feelings of anxiety and uncertainly, FDI: Feelings of depression and inadequacy FPS: Fatigue and physical symptoms, AS: Autonomic symptoms, (* p < 0.001).

## Discussion

We translated the PHRF-SCL (SF) into five international versions to create a multifaceted yet simple and convenient approach for evaluating the stress responses of foreign nationals residing in Japan. We verified the reliability and validity of the English, Chinese, Korean, Indonesian, and Vietnamese versions of the PHRF-SCL (SF) through a survey targeting participants aged from 18 to 64 years who were native speakers of the relevant languages. This study showed the possibility of using this measure for concise and multifaceted stress response assessment in countries and regions using these languages. It shows great promise as an indicator for assessing the mental health of foreign residents, which is overlooked in many cities around the world that host immigrants.

Each subscale had sufficient reliability. A good fit was confirmed by confirmatory factor analysis, with results at a level similar to that of results reported by Imazu et al. (GFI = 0.94, AGHI = 0.93, CFI = 0.91, and RMSEA = 0.052) for Japanese participants [5]. The severity classification obtained from the DASS 21 subscale scores was used to examine construct validity. Approximately half of the participants in the present study were classified as “normal” for all language versions and all subscales. Scores in the PHRF-SCL (SF) were significantly lower in the normal group than they were in the symptomatic groups (depression, anxiety, or stress) for all subscales, indicating construct validity to a large extent.

Intended for use with healthy participants, the PHRF-SCL (SF) is a scale created based on psychological and physical stress responses; half of the items relate to the latter [5]. In contrast, the DASS 21 aims to distinguish between depression and anxiety symptoms, and the physical reactions common to both are, therefore, excluded from the questions of the depression and anxiety scales [6]. Despite these differences in scale design, significant correlations were found between the PHRF-SCL(SF) and DASS 21, indicating concurrent validity.

Japan is attracting an increasingly diverse foreign resident population, representing more and more countries and regions of origin. The Japanese government encourages the provision of information intended for foreign nationals residing in and visiting Japan not only in their respective native languages but also in simplified Japanese (“Yasashii Nihongo”). Information translated into simplified Japanese is effective in the context of not only more widely communicating rules and services in daily life but also emergencies, such as evacuation information in the event of a disaster. However, there is a limit to the information that can be provided for all services in simplified Japanese. The Japanese Ministry of Health, Labour, and Welfare is working on publishing multilingual explanatory literature for foreign nationals and providing remote interpretation services for multilingual support; both of these services are intended for use in medical institutions [9]. Similar to medical care, when assessing mental and physical health, it is difficult to substitute terms with simplified Japanese; therefore, it is desirable to conduct such assessments in the subject’s native language.

The translated versions of the PHRF-SCL (SF) are appropriate and fit for the purposes of mental health screening and health education for foreign nationals in settings such as workplaces and schools. If an individual’s mental health can be accurately assessed in their native language, it will be possible to provide services to those needing support in their native language or in a simple manner. In areas where mental health measures for foreign residents are inadequate, the implementation of multilingual stress checks can be expected to broaden the range of people who can receive mental health support.

In this study, we deduced that a simple and effective evaluation of stress responses in healthy participants can be performed in multiple languages. Because the data for this study were collected through online surveys, the possibility of bias existing in the educational background or a sense of economic hardship existing that might affect stress responses in a manner that varies depending on the participants’ age and the country in which the survey was conducted cannot be excluded. Future surveys should consider the backgrounds of participants.

While a characteristic feature of the PHRF-SCL (SF) is its use of question items relating to stress responses in daily life, Imazu et al. [5] suggest that the Japanese version of the PHRF-SCL (SF) may help in the identification of persons with psychosomatic problems. The present study identified a high degree of correlation between the PHRF-SCL (SF) and the DASS 21, the purpose of which is to screen for symptoms of depression and anxiety. Therefore, in a patient-based study, we hope to verify the construct validity and clarify the relation between the multilingual versions of the PHRF-SCL(SF) and depression and anxiety scales.

## Conclusion

This study confirmed the reliability of the English, Chinese, Korean, Indonesian, and Vietnamese versions of the PHRF-SCL (SF), a scale used to assess stress conditions in healthy adults. The validity of these versions of PHRF- SCL(SF) was confirmed in two ways with the same scale. Although statistical studies are still needed, the results sufficiently substantiated that the scale was adequate for practical use. This scale is expected to contribute to the promotion of mental health services formultiple races and countries.

## Data Availability

The datasets generated and analyzed during the current study are not publicity available because no participant consent was received for the secondary use of their data, but they are available from the corresponding author on reasonable request.

## List of abbreviations

PHRF-SCL (SF): Public Health Research Foundation Stress Checklist Short Form
WHO: World Health Organization
DASS 21: Depression Anxiety Stress Scale 21
